# miR-223 and other miRNA's evaluation in chronic kidney disease: Innovative biomarkers and therapeutic tools

**DOI:** 10.1016/j.ncrna.2019.01.002

**Published:** 2019-01-23

**Authors:** Valérie Metzinger-Le Meuth, Laurent Metzinger

**Affiliations:** a INSERM U1148, Laboratory for Vascular Translational Science (LVTS), UFR SMBH, Université Paris 13-Sorbonne Paris Cité, 93017 Bobigny Cedex, France; b HEMATIM EA4666, C.U.R.S, Université de Picardie Jules Verne, 80025 Amiens Cedex 1, France

## Abstract

microRNAs (miRNAs) represent a recent breakthrough regarding gene expression regulation. They are instrumental players known to regulate post-transcriptional expression. miRNAs are short single stranded RNAs that base-pair with target mRNAs in specific regions mainly within their 3′ untranslated region. We know now that miRNAs are involved in kidney physiopathology. We outline in this review the recent discoveries made on the roles of miRNAs in cellular and animal models of kidney disease but also in patients suffering from chronic kidney disease, acute kidney injury and so forth. miRNAs are potential innovative biomarkers in nephrology, but before being used in daily clinical routine, their expression in large cohorts will have to be assessed, and an effort will have to be made to standardize measurement methods and to select the most suitable tissues and biofluids. In addition to a putative role as biomarkers, up- or down-regulating miRNAs is a novel therapeutic approach to cure kidney disorders. We discuss in this review recent methods that could be used to deliver miRNAs in a specific and suitable way in kidney and other organs damaged by kidney failure such as the cardiovascular system.

## 1 MiRNAs, a novel class of gene regulators

In the recent years, a great number of new non-coding RNAs (ncRNAs) has been discovered, bringing new lights on the gene regulation process [1]. Based on size, they are divided into either short noncoding RNA (<200 nucleotides including microRNA (miRNA), piwi RNA, snoRNA) or long noncoding RNA (lncRNA and circular RNAs, >200 nucleotides) [2]. miRNAs comprise a novel class of endogenous small (approx. 20 to 25 nucleotides) non-coding RNAs that negatively regulate gene expression via degradation or translational inhibition of their target mRNAs [3]. Thousands of miRNAs are now listed by dedicated internet databases such as mirBase, Tarbase, MicroRNA.org. miRNAs are first transcribed as a larger RNA product, Pri-miRNA, a few hundred to a few thousand nucleotides long, by the RNA polymerase II. They are then matured by a specific RNase III (Drosha) and its protein partner, DiGeorge syndrome critical region 8 (DGCR8) in the nucleus into a pre-miRNA hairpin of approx. 60–70 nt. This species is then exported into the cytoplasm by exportin where it is recognized and cleaved within its stems by the Dicer RNase III into a double stranded miRNA/miRNA* duplex (approx. 22 bp). One of the strands will be incorporated in the RNA-induced-silencing complex and become the mature miRNA. The other strand will most of the time be degraded quickly, or become a miRNA* and have its own biological effects. The RISC complex carries the mature miRNA to its target messenger RNAs, which results in gene silencing*.*

miRNAs bind mRNAs mostly in their 3′ untranslated region (UTR), and in some instances, the coding region or 5’UTR. The seed sequence, a 7-nt long region located most of the times between nucleotides 2–8 of the miRNA is the most important in complementary base-pairing between mRNA and miRNA. Mature miRNA sequences, especially the seed sequence, are conserved throughout evolution. miRNA pathways are conserved in the phylogeny, with a correlation between the number of miRNA genes, their expression levels, and the diversity of their targets. The remarkable conservation of the seed sequence is used to classify miRNA into families based on shared seed sequences by most miRNA target prediction algorithms. The softwares developed evaluate target mRNA and miRNA interactions by scoring (1) the complementarity between miRNA and mRNA 3′ UTR and (2) the degree of conservation of miRNA across species.

Outside of the seed region, several mismatches are common, explaining why one single miRNA can regulate the expression of multiple target genes. Indeed, a single miRNA can regulate multiple genes by its ability to hybridize with its mRNA targets as either perfect or imperfect complement. Conversely, one mRNA can be bound and regulated by several miRNAs. The 2000 miRNAs known to date can regulate up to 2/3rd of the human genome and impact multiple levels of developmental pathways, using extensive regulatory networks of gene expression. Mechanistically, miRNAs can act either by inhibiting translation of target messengers, or by inducing their degradation, and are thus posttranscriptional regulators. According to Bartel's team, most of the times mammalian miRNAs destabilize target mRNAs and thus decrease their numbers [4].

It is however increasingly evident that miRNAs act by other ways complementary to the canonical pathway. For example, we used a multi-omics approach that combined transcriptomics, proteomics and metabolomics to look at the way over-expression and inhibition of a given microRNA (here miR-223) impacts the whole of the genome response [5]. We found profound changes in the levels of proteins (52 significantly differently expressed), mRNAs (120 significantly differently expressed), and metabolites in order to identify genes involved in miR-223 regulation, with links to pathways including to the NF-kB pathway, histone acetylation, and ubiquitination. The majority of studies using transcriptomics assume that alterations in mRNA quantities and changes in protein expression are correlated, which is often not true [6,7]. In our hands, no gene was found to be deregulated by miR-223 in common between proteomics and transcriptomics. Our results seem thus to indicate that miR-223 changes the gene program of osteoclastogenesis and macrophage differentiation via the targeting of targets genes via mostly indirect mechanisms. As a result, changes in miR-223 expression alter the metabolic profile of cells, and may affect their apoptotic and proliferative state.

## 2 miRNAs are new players in the nephrology field

Chronic kidney disease (CKD), also called chronic kidney failure, is characterized by the gradual loss of kidney function. It is becoming increasingly evident that miRNAs are potential important players in the CKD field as they are expressed in the normal and pathological tissue and are involved in kidney diseases [8]. miRNAs can be detected in kidney as their expression is deregulated in pathological conditions. Aberrations of miRNA expression in renal fibrosis have been described in a recent meta-analysis that identified five up-regulated (miR-142–3p, miR-223–3p, miR-21–5p, miR-142–5p, miR-214–3p) and two down-regulated (miR-29c-3p, miR-200a-3p) miRNAs [9]. It is however apparent that cardiovascular complications are the leading cause of death in patients with CKD, so we will focus in this review on the influence of miRNAs on the cardiovascular side of CKD, and particularly vascular calcification. Cardio-vascular disease plays a capital role in CKD, as patients afflicted by later stages have a higher cardiovascular morbimortality [10,11] associated with vascular calcifications. Vascular calcification is a common complication in CKD, and is defined as a pathological deposition of calcium and phosphate in the vascular system [12], including intima and media, but can also be found in heart valves [13]. It is an active osteogenic process resembling bone formation that actually takes place in blood vessels [14].

Uremic toxins represent a complex mixture of compounds that accumulate in the serum of CKD patients and in turn accelerate vascular calcification, aging and organ dysfunctions. There is a direct correlation between the accumulation of uremic toxins and their biological and toxic effect in patients [15]. To assess this, we tested the effect of one of the most well-known uremic toxin inorganic phosphate in an *in vitro* model of vascular calcification: it consisted of human primary cultures of vascular smooth muscle cells (VSMCs) that acquired a pro-calcifying phenotype in presence of pathological levels of inorganic phosphate (Pi) [[16], [17], [18], [19]]. In this model, we found that Pi increased markedly the level of miR-223 [17]. We confirmed this *in vivo* in calcified aortas of CKD mice, as miR-223 levels increased. It is important to note that in the serum of CKD mice, seric miR-223 levels decreased (while levels increased in the aorta) [17]. This finding was confirmed in CKD patients [20]. We also found roles for miR-126, miR-143, miR-145 alongside miR-223: miR-126 expression increased in CKD and calcified aorta while miR-143 and miR-145 levels decreased in this calcified tissue [17]. It is known that supplementation in magnesium can decrease or even avoid the development of vascular calcification found in CKD [21], and we found that treating calcific VSMC with magnesium was able to revert at least partly the effect of Pi on miRNA expression in VSMCs [22]. Our combined results are thus in favor of a direct link between miRNA levels changes and uremic vascular toxicity. An intriguing hypothesis is that vascular calcification arises because osteoclast-like cells presence is limited in the vascular wall due to uremic toxins. The same factors promoting VSMC calcification could also inhibit osteoclastogenesis, *ie* the differentiation of monocytes/macrophages into osteoclast-like cells. In this light, we showed that Pi was responsible for a marked decrease in osteoclastogenesis in a model of osteoclastogenesis (RAW cells differentiated into osteoclasts), and that it was correlated with lower miR-223 levels in the cell [23]. Inhibiting miR-223 using an antimiR specific for this miRNA inhibited osteoclastogenesis as Pi did. Conversely, over-expressing miR-223 led to an enhancement of osteoclastogenesis. miR-223 modulated the expression of relevant target genes NFIA and RhoB but also of osteoclast marker genes and the Akt signaling pathway that in turn induced osteoclastogenesis [23]. These results were confirmed by measuring bone resorption activity in human peripheral blood mononuclear cells differentiated into osteoclasts - suggesting that a strategy to modulate miR-223 in CKD patients and activate osteoclasts in the vascular wall with the aim to reverse vascular calcifications might be useful in CKD*.* Conversely, Ji et al. [24] asserted that miR-223 regulates osteoclast differentiation by inhibiting NFIA expression. For this, miR-223 binds to specific sites within the promoter of NFIA and represses transcription by influencing epigenetic events.

Uremic toxins are also implicated in other cardiovascular complications in CKD. The serum concentration of certain heart-specific miRNAs seemed to be inversely related to renal function [10]. Levels of miR-223 were evaluated in a period of 4 years in a large cohort with respect to cardiovascular death outcome, and was found to be a predictor for cardiovascular death, and thus a potential biomarker with prognostic value [25]. Deregulation of cerebral blood flow and protection of the brain have been reported in CKD and are responsible for ischemic strokes and behavioral troubles. We quantified the expression of miR-17 and miR-126 in endothelial cells in cerebral arterioles of WT mice and of pathological models of CKD using RT-qPCR and showed that miR-17 and miR-126 are deregulated and can be used as new biological markers of cerebral troubles of patients in CKD [19].

## 3 miR-223 can affect gene expression levels in endothelium. The example of miR-223 target, RhoB

miR-223, which was first described as a modulator of hematopoietic lineage differentiation is also deregulated in several types of cancers and has an emerging role in inflammatory and metabolic disorders, particularly muscle diseases, type II diabetes, atherosclerosis and vascular calcification (eg Kim et al. [26], Guo et al. [23,27] recently reviewed in Ref. [28]).

Anglicheau et al. published that miR-223 levels were increased in kidney biopsies of patients suffering from progressive chronic renal failure compared to patients with stable CKD, indicating that miR-223 may have a role in the aggravation of renal dysfunction [29]. miR-223 was also found to be downregulated in pre-clinical and cellular models of pulmonary arterial hypertension (PAH). RhoB, a classic target of miR-223 [16], which was increased by hypoxia, and serum miR-223 levels were decreased in female patients with PAH associated with congenital heart disease [30].

In this review we postulate that RhoB could be an interesting target to modulate using miR-223, as there is a strong link among endothelial contractility, vessel stiffening and vascular disease, which is controlled by Rho-driven actomyosin contractility [31]. Indeed, the Rho guanosine triphosphatase family has been identified as a key regulator of angiogenesis and the deletion of RhoB was reported to increase endothelial apoptosis and impair endothelial cell migration and tube formation. Interestingly, the overexpression of RhoB was demonstrated to induce endothelial permeability and growth [32]. As miR-223 inhibition induces an increase of RhoB expression, an effect on endothelial cells is possible. A few studies were realized about miR-223 effect on endothelial cells because miR-223 is expressed in native endothelial cells and is rapidly downregulated by cell isolation and cultures, so miR-223 is not studied in cell lines [33]. Shi et al. [33] demonstrated that miR-223 is antiangiogenic and prevents the proliferation of freshly isolated endothelial cells by targeting β1integrin and also growth factor signaling in these endothelial cells. It seems that miR-223 may be necessary for the maintenance of endothelial cell quiescence [33]. So, one can propose that an inhibition of miR-223 levels could prevent endothelial dysfunction by at least partly increasing RhoB.

## 4 miRNAs are future biomarkers of CKD

Nowadays, glomerular filtration rate remains the most used and optimal marker of renal function. That said, taking an exact measure in clinical routine is complicated so it is estimated from creatinine seric levels, and so lacks sensitivity for the early detection of CKD [34]. A number of potential biomarkers such as urinary connective tissue growth factor, urinary and serum neutrophil gelatinase-associated lipocalin, urinary liver fatty acid-binding protein, urinary N-acetyl-β-d-glucosaminidase, serum apolipoprotein A-IV, serum and urinary Kidney injury molecule 1 or serum fibroblast growth factor 23 have been described recently (reviewed in Ref. [34]). None has been validated in clinical practice. miRNAs have been put into focus as they represent innovative biomarkers in medical biology [35], since Mitchell et al. [36] demonstrated for the first time that they are detectable in human blood. In this liquid tissue, they are protected from RNases as they are present in cell-derived membrane vesicles, HDL lipoproteins or in ribonucleoprotein complexes with protein partner Argonaute 2 [37]. An advantage of miRNA is that they are stable in serum, and meet the sensitivity, specificity and reproducibility criteria required of a non-invasive biomarker [35].

The general consensus is that, most of the time, miRNA levels in general decrease as CKD progresses [38]. Hüttenhofer and Mayer in their review [39] report that clinical data on renal clearance of miRNAs are sparse, but in case of acute myocardial infarction it has been suggested that a change in glomerular filtration rate affects serum levels of miRNAs. On the other hand, Neal et al. [38], report that total miRNA levels and levels of five specific miRNAs (−16, −21, 155, −210 and −638) all are reduced in patients with advanced renal failure when compared with controls. In a cohort of 90 patients at CKD stages 3-5D, Chen et al. showed that miR-125b, miR-145 and miR-155 levels decrease compare to normal patients [40]. We also showed in a murine model of CKD that seric miR-223 decrease and this was recently confirmed in CKD stage 4 and 5 patients [20]. Interestingly, in these Austrian transplanted patients, this deregulation alleviated after renal transplantation.

## 5 The importance of the reliability and reproducibility of the measurement of seric miRNAs in routine clinical practice

**Fig. 1:**
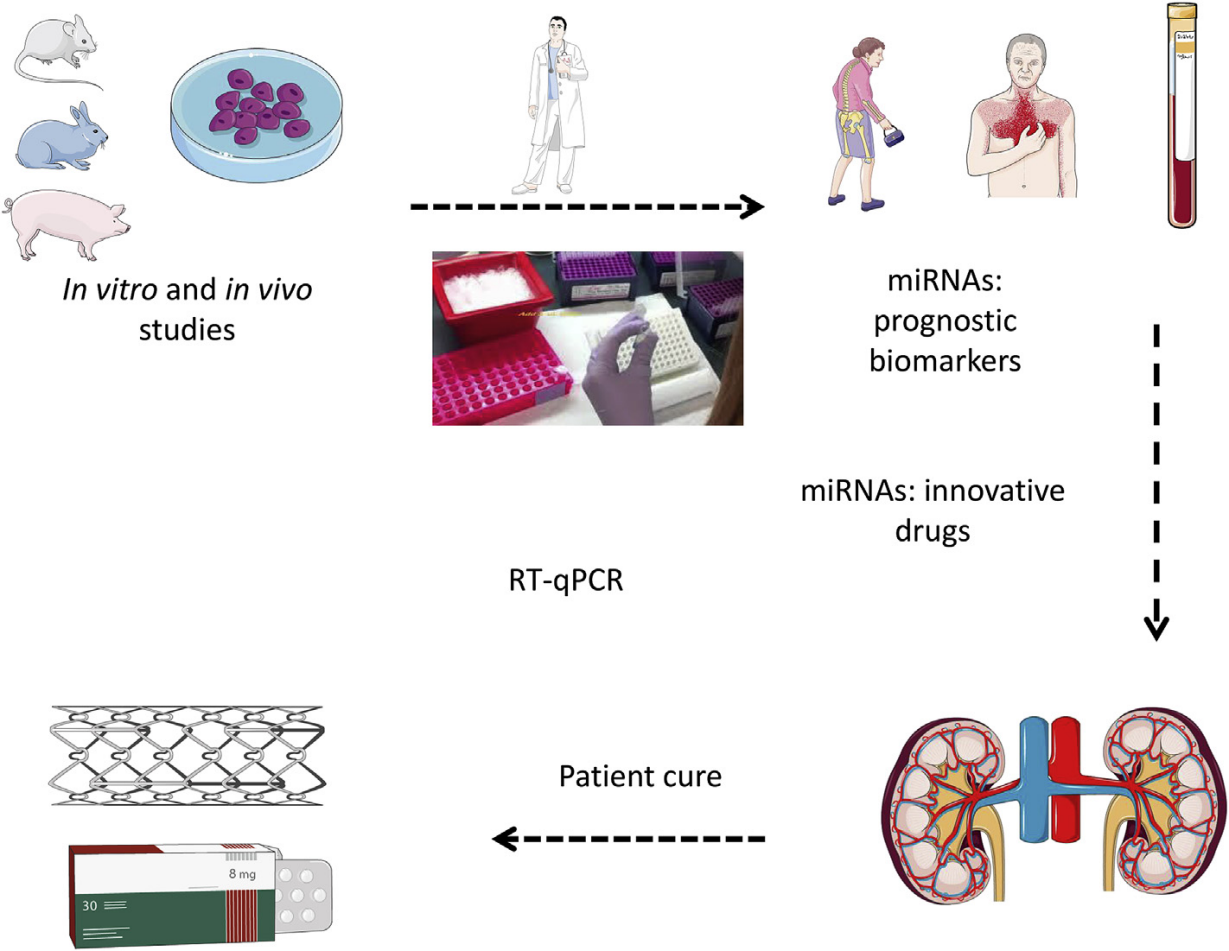
The potential of miRNAs to assess and cure renal disorders: Preclinical models are useful to screen miRNAs implicated in nephrological diseases. miRNAs can also be used as innovative therapeutic approaches to improve kidney pathology.

We are far from an accurate and useful miRNA signature for the diagnostic or prognosis of CKD. One of the main obstacles to overcome is to quantify miRNAs’ expression levels, as most of the labs use different techniques, leading to poor reproducibility [35]. Real-time PCR is acceptable for the measurement of a small number of miRNAs as it is a swift technique, easily transferable into routine practice (Fig. 1). The community will have to decide which control to use to normalize expression levels of circulating miRNAs: endogenous circulating miRNAs (eg U6 and miR-1249) can be used; but their expression often varies in pathological conditions [35]. Standardization is very important. so one can add a precise amount of exogenous miRNA (e.g. synthetic *Caenorhabditis elegans* miR-39) to avoid experimental bias [35]. Although RT-qPCR is quick in the standard of a molecular biology lab, it can be cumbersome and long when considered in the light of standard clinical routine. Replacement techniques must be considered and are developed at the moment. For example, nanobiophotonic detection that involves Förster resonance energy transfer (FRET) after the excitation of two fluorophores [41], or a method for the electrochemical detection of miRNAs in urine samples [42].

## 6 miRNAs as new therapeutic targets in restenosis and other vascular dysfunctions

miRNAs are important keys to regulate the gene response to various pathophysiological stimuli and have thus potential therapeutic impact [1].

The overexpression of some miRNAs could be the result of an adaptative response in order by instance, to decrease cell proliferation. miR-424 and its rat ortholog miR-322 were up-regulated in proliferative VSMC and after vascular injury with an anti-proliferative and an anti-differentiation effect. This seemed to be an adaptive response overwhelmed in pathological situations as the overexpression of miR424/322 induced by an adenovirus in injured rat carotid arteries demonstrated a protective effect against restenosis [43]. Other miRNAs are down-regulated after vascular injury. miR-145 and miR-143 are two miRNAs from the same cluster which are up-regulated in differentiated VSMC. They participate to the maintenance of the VSMC contractile phenotype and their over-expression prevents neointimal hyperplasia. Their expression is reduced when VSMC are activated after a vascular injury or stress [[44], [45], [46]].

**Fig. 2:**
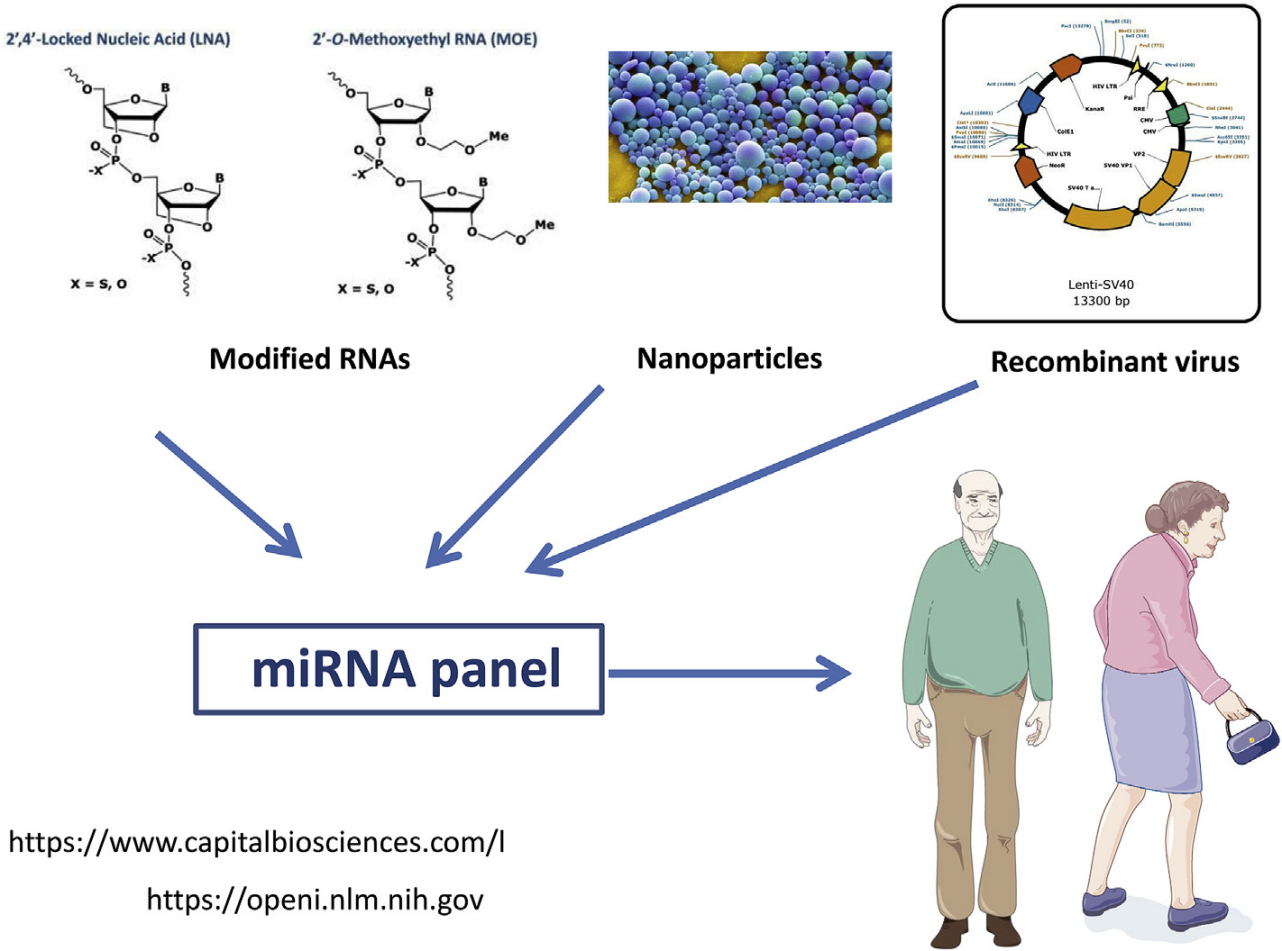
Altering a miRNA expression to counteract pathologies in ageing and diabetic patients, prone to develop CKD. The expression levels of a panel of adapted miRNAs can be altered according to the clinical context. Various approaches are at the moment developed for this purpose. Second generation gene therapy vectors, such as lentiviruses can be used, to produce RNA constructs *in situ*. Gold or iron-nanoparticles can also be used to deliver RNAs. Finally, the RNAs can be chemically modified to increase their stability. LNA: locked nucleic acid, MOE: 2′—O-(2-Methoxyethyl)- oligoribonucleotide.

These results indicate that miRNAs precursors (to over-express) and miRNAs inhibitors (to inhibit) have to be delivered in a given time- and tissue-specific manner, in order to get an efficient therapeutic response in CKD (Fig. 1). Different approaches of injured arterial wall treatment have used systemic, polymer-based particles or local, modified stents to induce vascular regeneration in CKD [47,48]. The use of antiproliferative drugs such as placlitaxel helped to reduce but did not completely inhibit the restenosis phenomenon [49]. Drug eluting stents reduced in-stent restenosis by their ability to release an anti-proliferative drug but the risks of inflammation and/or late thrombosis increases. The development of new therapies for restenosis is still a challenge and miRNAs, which have a key role in the regulation of several cardiovascular pathologies, are actually investigated as potential biomarkers and therapeutic targets for this phenomenon [18,45]. Other targets such as protein SERCA2 are under development to target restenosis [50], but miR-223 could join other miRNAs such as miR-24 [51] and miR-424/322 [43] to develop innovative therapeutic strategies. The cellular mechanisms linked to restenosis are not completely understood and the cellular composition of neointima is still a matter of controversy. Neointima formation seemed to be induced by the removing of endothelial cells (ECs) following vascular lesion which could provoke VSMC proliferation and migration towards vascular lumen and leukocytes infiltration [52]. According to literature, the myofibroblasts and VSMC cells from the adventice and the vascular wall could be implicated in the neointima formation process. Nevertheless, VSMC are the most abundant cellular type in neointima hyperplasia [45,53]. Microvesicles, exosomes, lipoproteins are able to carry functional miRNAs in conjunction with lipids, proteins, mRNAs, ncRNAs … as cargos to target cells so we can use these naturally occurring nanomaterials already present in the human body [32], to deliver functional miRNAs [54]. Fig. 2 summarizes various technological ways that are at the moment developed to alter miRNA expression. Nanotechnology can be used: new vehicles of gold nanoparticles of 13 nm, functionalized with monolayers of alkylthiol-modified RNA molecules [55] are able to transfect a variety of cells without the aid of co-carriers for up to 24 h and deliver endogenous miRNAs *in situ* [55]. Viral vectors have emerged as attractive vehicles for the delivery of transgenes to specific cell types and represent another way to produce RNA interfering with target miRNA expression into cells [56]. The absence of adeno-associated virus (AAV) pathogenicity in humans makes them an attractive solution. Another class of second generation vector is lentiviruses. They are often considered superior to AAV as they are able to transduce dividing and nondividing cells, but as they represent a subgroup of the *Retroviridae* family that includes the human immunodeficiency virus type 1, safety issues are a concern [56]. Chemical modifications of RNA represent however the most used strategy in the various clinical trials ongoing at the moment [57]. The main approaches are to use single-stranded antisense oligonucleotides and duplex RNAs. Since RNAs are prone to degradation, poorly distributed to cells and have unfavourable pharmacokinetic properties, they are chemically modified to produce locked nucleic acids (Fig. 2) or other modified RNAs (reviewed in [57]. These chemical modifications are now powerful enough to induce long lasting effects in the latest clinical trials [57], and despite the observation of adverse events, one can postulate that RNA-based therapies will be used in the future to deliver miRNAs in diseased tissues, such as vessels and kidney in CKD patients.

CRISPR/cas9 system is another tool than can be used in the miRNA field, as specific CRISPR/cas9 constructs can be used to target selected sites to abolish the long term expression of miRNAs [58].

## 7 Concluding remarks

miRNAs are potential new solutions in the nephrology field in order to develop innovative biomarkers for the early diagnosis and prognosis of patients afflicted with kidney disease*.* Modulating miRNA levels could be a solution in the very next future for therapies of kidney diseases among a world population of ageing and diabetic patients, prone to develop CKD (Fig. 1, Fig. 2).
